# Genomic Analysis of IgG Antibody Response to Common Pathogens in Commercial Sows in Health-Challenged Herds

**DOI:** 10.3389/fgene.2020.593804

**Published:** 2020-10-23

**Authors:** Leticia P. Sanglard, PigGen Canada, Benny E. Mote, Philip Willson, John C. S. Harding, Graham S. Plastow, Jack C. M. Dekkers, Nick V. L. Serão

**Affiliations:** They participated in project and protocol development and implementation, coordinated the sources of sows and collection of associated data, and contributed to the project through regular discussions during execution of the gilt acclimation project: Mr. D. Vandenbroek and Mr. B. DeVries, Alliance Genetics Canada, St. Thomas, ON, Canada; Dr. N. Dion and Ms. S. Blanchette, AlphaGene, Saint-Hyacinthe, QC, Canada; Dr. T. Rathje, DNA Genetics, Columbus, NE, United States; Mr. M. Duggan, FastGenetics, Saskatoon, SK, Canada; Dr. R. Kemp, Genesus, London, ON, Canada; Dr. P. Charagu, Hypor, Regina, SK, Canada; and Dr. P. Mathur, Topigs Norsvin, Helvoirt, Netherlands.; ^1^Department of Animal Science, Iowa State University, Ames, IA, United States; ^2^Department of Animal Science, University of Nebraska–Lincoln, Lincoln, NE, United States; ^3^Canadian Centre for Health and Safety in Agriculture, University of Saskatchewan, Saskatoon, SK, Canada; ^4^Department of Large Animal Clinical Sciences, University of Saskatchewan, Saskatoon, SK, Canada; ^5^Department of Agricultural, Food and Nutritional Science, University of Alberta, Edmonton, AB, Canada

**Keywords:** antibody response, genetic correlation, GWAS, heritability, infectious pathogens

## Abstract

Losses due to infectious diseases are one of the main factors affecting productivity in the swine industry, motivating the investigation of disease resilience-related traits for genetic selection. However, these traits are not expected to be expressed in the nucleus herds, where selection is performed. One alternative is to use information from the commercial level to identify and select nucleus animals genetically superior for coping with pathogen challenges. In this study, we analyzed the genetic basis of antibody (Ab) response to common infectious pathogens in health-challenged commercial swine herds as potential indicator traits for disease resilience, including Ab response to influenza A virus of swine (IAV), *Mycoplasma hyopneumoniae* (MH), porcine circovirus (PCV2), and *Actinobacillus pleuropneumoniae* (APP; different serotypes). Ab response was measured in blood at entry into gilt rearing, post-acclimation (∼40 days after entering the commercial herd), and parities 1 and 2. Heritability estimates for Ab response to IAV, MH, and PCV2 ranged from 0 to 0.76. Ab response to APP ranged from 0 to 0.40. The genetic correlation (r_*G*_) of Ab response to IAV with MH, PCV2, PRRSV, and APP_mean_ (average Ab responses for all serotypes of APP) were positive (>0.29) at entry. APP_mean_ was negatively correlated with PCV2 and MH at entry and parity 2 but positively correlated with MH at post-acclimation and parity 1. Genomic regions associated with Ab response to different APP serotypes were identified on 13 chromosomes. The region on chromosome 14 (2 Mb) was associated with several serotypes of APP, explaining up to 4.3% of the genetic variance of Ab to APP7 at entry. In general, genomic prediction accuracies for Ab response were low to moderate, except average Ab response to all infectious pathogens evaluated. These results suggest that genetic selection of Ab response in commercial sows is possible, but with variable success depending on the trait and the time-point of collection. Future work is needed to determine genetic correlations of Ab response with disease resilience, reproductive performance, and other production traits.

## Introduction

Infectious diseases are well known to cause productivity losses in the swine industry (Lee et al., 2012; Lewis et al., 2007), motivating the investigation of traits related to disease resilience for genetic selection. It has been shown that there is genetic variation in total antibody (Ab) response to swine pathogens, such as porcine reproductive and respiratory syndrome (PRRS) virus (PRRSV) (Serão et al., 2014; Hess et al., 2018; Abella et al., 2019). Selection of more resilient animals could decrease the losses caused by the decreased performance of animals exposed to pathogens.

A common limitation for genetic selection of improved host response to infectious pathogens is that these traits are not expected to be expressed in the nucleus, where selection is performed, because of high biosecurity (Faust et al., 1993). Disease traits are usually expressed at the commercial level, such as during the acclimation or introduction period of gilts into a commercial herd, when they are exposed to several pathogens (Serão et al., 2016). Therefore, one alternative would be to identify genetically superior animals in their ability to overcome the pathogen challenge at the commercial level and use this information to select animals at the nucleus level.

The interest for improved performance in the presence of a wide range of infectious pathogens has led to several studies showing genetic variation for resilience-related traits in livestock (Clapperton et al., 2009; Engle et al., 2014; Serão et al., 2014). More specifically, it has been shown that host genetics plays a role in differences in Ab response in swine (Flori et al., 2011). For instance, pigs selected for a higher immune response after 8 generations presented higher Ab response to various antigens and grew faster than pigs with a lower immune response (Mallard et al., 1998). For PRRSV, the major viral pathogen impacting swine production, moderate to high heritability (h^2^ = 0.38–0.46) has been reported for Ab response to this disease in commercial gilts (Serão et al., 2016; Sanglard et al., 2020). Dunkelberger et al. (2017) reported a high h^2^ for PRRS viral load (0.61) but not for porcine circovirus type 2 (PCV2; 0.09). In their study, pigs were vaccinated to PRRSV and co-infected with field strains of both viruses. However, other common pathogens, such as influenza A virus of swine (IAV), *Mycoplasma hyopneumoniae* (MH), and *Actinobacillus pleuropneumoniae* (APP) are also involved in the porcine respiratory disease complex (Thacker et al., 1999, 2001; Bates et al., 2009), which is one of the main causes of economic losses in the swine industry. Nonetheless, host-genomic studies of animals exposed to these pathogens are not available in the literature.

Studies have shown that genomic selection using estimates of marker effects on crossbred animals from the commercial level is a good alternative to increase response to selection and, consequently, the performance of commercial animals (Dekkers, 2007). Serão et al. (2016) and Sanglard et al. (2020) showed that Ab response to PRRSV associated with genomic information collected at the commercial level can be used to predict breeding values for Ab response to PRRSV with moderate to high accuracy in crossbred sows. Moderate accuracy of prediction of breeding values for Ab response has also been reported for Newcastle disease and avian influenza virus in chickens (Liu et al., 2014). These results support the possibility of using Ab response for selection for resilience in commercial animals. However, genomic analyses of many common infectious pathogens in pigs are lacking in the literature. Therefore, the objective of this study was to investigate the genetic basis of Ab response to common infectious pathogens in swine production in replacement gilts during acclimation raised in commercial farms [same population as described in Serão et al. (2016)] by (1) estimation of co-variance components of Ab response; (2) identification of quantitative trait loci (QTL) for Ab response; and (3) assessment of the genomic prediction accuracies for Ab response. In order to maximize the robustness and relevance of results to the field, the data collected in this study was by design highly variable, representing data from 23 commercial farms across Canada, with different gilt acclimation and vaccination protocols.

## Materials and Methods

All procedures for the experiment were performed according to the Canadian Council on Animal Care (2020) base on the Guide to the Care and Use of Experimental Animals, vol. 1, Olfert ED, Cross BM (Ottawa, ON, Canada).

### Animals

The datasets used in this study were provided by a consortium of pig breeding companies (genetic suppliers) that operate in Canada (PigGen Canada)^1^. The data included 2,848 commercial F1 (Landrace × Large White) replacement gilts sourced from 17 high-health multipliers from seven breeding companies, all members of PigGen Canada. Replacement gilts were introduced to 23 commercial farms with historical occurrences of natural disease challenges, following the standard acclimation procedures of each farm, including each farm’s routine vaccination protocols, in contemporary groups (CG) of 10 to 63 animals (27 ± 15 animals per CG), with a total of 107 CG. The summarized information of the vaccination protocols provided by each farm is provided in Table 1. Time of vaccination differed between farms and occurred during entry to the commercial level, during quarantine, during acclimation, in mid-lactation, after weaning, or at alternate parities. Records on administration and dates of vaccination were not available. There were also no records on whether animals were naturally infected with any of those pathogens. A full description of the dataset can be found in Serão et al. (2016).

**TABLE 1:** Counts and reported vaccination protocols^1^ for contemporary groups (CG) by genetic supplier (GS).

**GS**	**MH**	**CH**	**CG (n)**	**N**	**Qt**	**PRRSV Vx**	**IAV Vx**	**MH Vx**
1	1	1	4	134	Yes	Yes	Yes	Yes
		2	3	99	Yes	Yes	Yes	No
	2	3	6	121	Yes	No	No	Yes
	3	4	5	110	Yes	Yes	Yes	Yes
2	4	5	9	120	No	Yes	No	Yes
	5	6	5	90	No	Yes	Yes	Yes
		7	4	47	Yes	No	No	No
	6	8	4	83	Yes	No	No	Yes
3	7	9	9	417	Yes	Yes	Yes	Yes
	8	10	3	120	Yes	No	Yes	Yes
4	9	11	4	133	Yes	No	Yes	Yes
		12	5	92	Yes	No	No	Yes
	10	13	5	150	Yes	No	Yes	Yes
5	11	14	3	101	Yes	Yes	Yes	Yes
		15	4	97	No	No	No	Yes
	12	16	5	131	No	Yes	Yes	Yes
	13	17	4	120	Yes	Yes	Yes	Yes
6	14	18	7	174	No	Yes	Yes	Yes
		19	2	50	No	Yes	Yes	No
	15	20	3	75	No	Yes	Yes	Yes
		21	3	74	Yes	Yes	Yes	Yes
7	16	22	4	159	Yes	No	No	No
	17	23	4	151	Yes	No	No	Yes

*^1^Pathogens potentially vaccinated for at each CG; GS, recoded ID for genetic supplier; MH, recoded ID for multiplier herd; CH, recoded ID for commercial herd; CG (n), number of contemporary groups; N, number of gilts per CG; Qt, quarantine, PRRSV Vx, CG vaccinated to Porcine Reproductive Respiratory Syndrome Virus; IAV Vx, CG vaccinated to Influenza A Virus; MH Vx, CG vaccinated to *Mycoplasma hyopneumoniae*.*

### Phenotypic Data

Blood samples were collected from all replacement gilts at four time-points: when entering the commercial herd (Entry), after the acclimation period (Post-acclimation), and during parity 1 (P1), and parity 2 (P2). The average time (± standard deviation) between Entry and Post-acclimation sampling was 40.8 ± 16.3 days, ranging from 29 to 88 days. Sample collection for P1 and P2 occurred between farrowing and weaning, but the exact date of collection was not available. Animals were not deliberately infected with any of the pathogens in the study; therefore, the level of exposure (if present) to these antigens was unknown and was likely variable, which further supports this study as a model for evaluating the overall genetic basis of response to pathogens in commercial swine populations.

Antibody response to PRRSV, IAV, MH, PCV2, and 8 serotypes of APP (APP1, 2, 3, 5, 7, 10, 12, and 13) were measured as sample-to-positive (S/P; PRRSV, MH, PCV2, and APP) or sample-to-negative (S/N; for IAV only) ratios. Antibody measurements were performed using ELISA (IDEXX PRRS X3, IDEXX Laboratories Inc., Westbrook, United States) for PRRSV, LC-LPS ELISA, developed by the Groupe de Recherche sur les Maladies Infectieuses en Production Animale (GREMIP; Université de Montréal, Montreal, Canada) for all serotypes of APP, IDEXX ELISA for MH; IDEXX Influenza A virus Ab test kit^®^ for IAV, and INgezim CIRCO IgG^®^ for PCV2. All analyses were performed at GREMIP. Since antibody response to IAV was the only pathogen measured in the opposite direction (S/N instead of S/P), we recalculate this measurement as S/P for analyses to facilitate the interpretation of the results. Two summaries of Ab response traits were also created: (1) APP_mean_, as the mean of S/P for all APP serotype; and (2) MEAN, as the mean of standardized Ab response (S/P ratio divided by the standard deviation) to all infectious pathogens, to summarize the overall Ab response.

Following Serão et al. (2016), five seroconverted datasets (SCD) were created for each time point (Entry, Post-acclimation, P1, and P2) and each pathogen (IAV, MH, PCV2, and APP) based on ≥0, ≥25, ≥50, ≥75, and 100% of seroconverted animals within a CG. For seroconversion, the following diagnostic thresholds were used: S/P ≥ 0.4 (MH, APP, and PRRSV), S/N ≤ 0.6 (IAV), and S/P > 0 (PCV2). Each pathogen at each time with a proportion of positive animals was considered a separate trait. The numbers of animals and mean Ab responses for each dataset are presented in Table 2. Datasets with less than 500 animals were not analyzed.

**TABLE 2:** Number of individuals and mean of antibody response across pathogens^1^, time points, and seropositive (%) datasets.

**Traits^2^**	**%^3,4^**	**Entry**		**Post-acclimation**		**Parity 1**		**Parity 2**	
		**N^4^ (positive)**	**Mean (SD)**	**N^5^ (positive)**	**Mean (SD)**	**N^5^ (positive)**	**Mean (SD)**	**N^5^ (positive)**	**Mean (SD)**
IAV	0	2478 (0.49)	0.65 (0.34)	2354 (0.68)	0.51 (0.32)	1968 (0.82)	0.37 (0.29)	1280 (0.88)	0.30 (0.24)
	25	1537 (0.76)	0.47 (0.28)	1907 (0.83)	0.41 (0.26)	1814 (0.88)	0.33 (0.24)	1220 (0.92)	0.27 (0.2)
	50	1351 (0.82)	0.43 (0.26)	1786 (0.86)	0.40 (0.25)	1693 (0.92)	0.30 (0.21)	1220 (0.92)	0.27 (0.2)
	75	877 (0.90)	0.37 (0.22)	1463 (0.90)	0.37 (0.24)	1543 (0.94)	0.28 (0.2)	1132 (0.94)	0.26 (0.19)
MH	0	2479 (0.37)	0.50 (0.62)	2355 (0.65)	0.87 (0.73)	1969 (0.78)	0.97 (0.65)	1280 (0.77)	1.02 (0.67)
	25	1147 (0.76)	0.95 (0.64)	1927 (0.78)	1.04 (0.70)	1684 (0.90)	1.12 (0.58)	1081 (0.90)	1.18 (0.60)
	50	935 (0.84)	1.07 (0.63)	1643 (0.85)	1.15 (0.69)	1637 (0.92)	1.14 (0.57)	1023 (0.93)	1.22 (0.58)
	75	656 (0.94)	1.29 (0.59)	1074 (0.96)	1.41 (0.64)	1410 (0.95)	1.22 (0.55)	946 (0.95)	1.26 (0.56)
	100	–		564 (1.00)	1.73 (0.56)	622 (1.00)	1.49 (0.52)	503 (1.00)	1.48 (0.51)
PCV2	0	2387 (0.84)	2967 (7823)	2329 (0.94)	9121 (26210)	1912 (0.94)	5239 (22556)	1257 (0.97)	2461 (9776)
	25	2346 (0.85)	3017 (7881)	2292 (0.95)	9268 (26395)	1912 (0.94)	5239 (22556)	1257 (0.97)	2461 (9776)
	50	2052 (0.91)	3403 (8347)	2202 (0.97)	9636 (26865)	1861 (0.96)	5374 (22847)	1257 (0.97)	2461 (9776)
	75	1782 (0.95)	3847 (8866)	2094 (0.99)	10056 (27458)	1772 (0.97)	5619 (23387)	1239 (0.98)	2492 (9843)
	100	955 (1.00)	5471 (11596)	1760 (1.00)	11703 (29639)	1221 (1.00)	7592 (27725)	908 (1.00)	3140 (11375)
PRRS	0	2454 (0.03)	0.07 (0.24)	2342 (0.81)	1.19 (0.72)	2022 (0.61)	0.94 (0.9)	1378 (0.56)	0.78 (0.79)
	25	–	–	2053 (0.93)	1.36 (0.61)	1713 (0.69)	1.05 (0.9)	984 (0.72)	0.98 (0.8)
	50	–	–	1886 (0.95)	1.4 (0.57)	1020 (0.80)	1.25 (0.93)	549 (0.84)	1.18 (0.81)
	75	–	–	808 (0.98)	1.45 (0.53)	–	–	–	–
APP1	0	2478 (0.06)	0.25 (0.17)	2354 (0.08)	0.29 (0.17)	1969 (0.05)	0.26 (0.08)	1280 (0.03)	0.25 (0.06)
APP2	0	2479 (0.04)	0.23 (0.08)	2354 (0.09)	0.27 (0.09)	1968 (0.11)	0.26 (0.11)	1280 (0.13)	0.25 (0.12)
APP3	0	2478 (0.01)	0.26 (0.05)	2354 (0.03)	0.28 (0.06)	1968 (0.14)	0.32 (0.15)	1280 (0.15)	0.31 (0.16)
APP5	0	2477 (0.02)	0.22 (0.06)	2354 (0.02)	0.24 (0.06)	1968 (0.04)	0.24 (0.08)	1280 (0.04)	0.23 (0.08)
APP7	0	2479 (0.01)	0.16 (0.04)	2354 (0.01)	0.17 (0.05)	1968 (0.10)	0.23 (0.16)	1280 (0.12)	0.24 (0.18)
APP10	0	2478 (< 0.01)	0.19 (0.05)	2354 (< 0.01)	0.2 (0.05)	1968 (0.02)	0.23 (0.08)	1280 (0.03)	0.22 (0.07)
APP12	0	2478 (0.03)	0.21 (0.08)	2354 (0.03)	0.24 (0.09)	1968 (0.21)	0.32 (0.19)	1280 (0.18)	0.31 (0.19)
APP13	0	2478 (0.01)	0.23 (0.05)	2354 (0.01)	0.24 (0.05)	1967 (0.04)	0.25 (0.09)	1281 (0.04)	0.26 (0.08)
APP_mean_	0	2479 (0.04)	0.22 (0.04)	2354 (0.04)	0.24 (0.05)	1969 (0.12)	0.26 (0.07)	1281 (0.05)	0.26 (0.09)
MEAN	0	2505	2.57 (0.59)	2364	2.68 (0.49)	2020	1.96 (0.52)	2048	2.49 (1.05)

*^1^Antibody response to Porcine Reproductive and Respiratory Syndrome is not being shown as it has been previously published by Serão et al. (2016). ^2^Traits: IAV, antibody response to influenza A virus; MH, antibody response to *Mycoplasma hyopneumoniae*; PCV2, antibody response to porcine circovirus type 2; APP, antibody response to *Actinobacillus pleuropneumoniae* from different serotypes (represented by the different numbers; APP_mean_, mean of antibody response to all serotypes of APP; MEAN, mean of standardized antibody response to all infectious pathogens. ^3^Minimum percentage of seropositive animals within a contemporary group (% seroconverted data). ^4^% seroconverted data were used when the total number of animals were >500. ^5^N, Total number of animals (proportion of positive animals based on the diagnostic thresholds of: SIV ≤ 0.6; MH, APP, and PRRS ≥ 0.4; PCV2 > 0).*

### Genotypic Data

A total of 316 animals were genotyped with the Illumina PorcineSNP BeadChip (Illumina Inc., San Diego, United States) at Delta Genomics (Livestock Gentec, Edmonton, Canada), of which 48, 1710, and 1857 were genotyped using versions 60 K v.2, 60 K v.2B, and 80 K, respectively (Illumina Inc., San Diego, United States). These versions include 62163, 61565, and 68528 single-nucleotide polymorphisms (SNP), respectively. A total of 42145 SNP was common to all three versions, and 38191 SNP that passed quality controls were used for the genomic analyses, based on gene call (GC) score >0.5, animal call rate of 80%, and genotype call rate of 99.48%. GC scores measure the quality of the genotyping call for each genotyped SNP within an animal. Of the 3516 genotyped animals, 668 were parents of the gilts and did not have Ab response phenotypes. Still, we kept their genotype information in the dataset to make use of their genomic relationships. A full description of the genotypic data can be found in Serão et al. (2016).

### Genetic Parameters

An animal model with a genomic relationship matrix (GRM) from the first method described by VanRaden (2008) was used to estimate co-variance parameters using the following model:

$$y_{i\,j}=\mu +C\,G_{i}+u_{i\,j}+e_{i\,j}$$

where *y*_*ij*_ is the phenotype of the *j*^*th*^ individual of the *i*^*th*^ *CG*; μis the intercept; *CG*_*i*_is the effect of the *i*^*th*^ level of the fixed effect of *CG*; *u*_*ij*_is the breeding value of the *j*^*th*^ individual of the *i*^*th*^ *CG*, with$u∼N\,\left(0,\mathrm{GRM}\,\sigma_{u}^{2}\right)$, where ***GRM*** is the genomic relationships matrix based on 38191 SNP and 3516 individuals, with SNP genotypes coded as 0/1/2 and averaged and centered within multiplier herd; and *e*_*ij*_ is the random residual effect, with$e∼N\,\left(0,I\,\sigma_{e}^{2}\right)$, where ***I*** is the identity matrix. The ***GRM*** was created separately for pigs from each breeding company, and relationships between breeding companies were assumed to be zero. The fixed effect of *CG* was included in the model to account for environmental effects due to the farms and other possible environmental effects confounded within the farms (i.e., the timing of PRRSV exposure, if occurred), and not for comparisons between CG.

Bivariate analyses were performed between Ab response to two pathogens within a time-point, and between two time-points for the same pathogen. Co-variance components were estimated for each of the %SCD and were used to estimate heritabilities (h^2^) and genetic correlations(*r*_*G*_). The same fixed and random effects as used for the univariate model were also used for the bivariate analyses.

### Genome-Wide Association Studies (GWAS)

Genome-wide association studies (GWAS) were performed using Bayesian variable selection methods (Habier et al., 2011) using GenSel 4.4 (Fernando and Garrick, 2009). The model used in these analyses included an intercept, the fixed effect of *CG*, and the random allele substitution effects of SNP. First, a BayesC0 analysis, a method that fits all SNPs simultaneously in the model, assuming each variance across SNPs, was performed to estimate the variance components for subsequent analyses. Then, BayesCπ was used to estimate the proportion of SNP with zero effect (π). The estimate of π was 0.99 for all datasets. The final GWAS were based on the BayesB method, with π equal to 0.99. One-Mb SNP windows that explained at least 1% of total genetic variance explained by the markers (TGVM) and that had a posterior probability of inclusion (PPI) greater than 0.7 (Garrick and Fernando, 2013) were considered significantly associated with the trait analyzed. The order of the SNP was based on the *Sus scrofa* 11.1 assembly. Candidate genes within 1-Mb in each direction of the identified SNP were identified using Ensembl BioMart (Kinsella et al., 2011).

### Genomic Prediction

Genomic prediction was performed using BayesC0, BayesB, and BayesC (Habier et al., 2011). Analyses were performed for each trait and for each %SCD using the same models as described for GWAS in GenSel 4.4 (Fernando and Garrick, 2009). Seven-fold cross-validation was used, in which data from six breeding companies were used for training and data from the remaining breeding company for validation. This was repeated seven times until all breeding companies were used once for validation. Thus, the relationships between folds (i.e., between genetic backgrounds) were decreased and those within folds were increased. These analyses were performed for each dataset. Accuracy of genomic prediction (AGP) was defined as the correlation between genomic estimated breeding values and phenotypes adjusted for estimates of fixed effects divided by the square root of the estimate of h^2^ using the whole dataset. For the seven-fold cross-validation, the accuracy was weighted by the number of individuals in the validation dataset.

## Results

### Phenotypic Data

**FIGURE 1:**
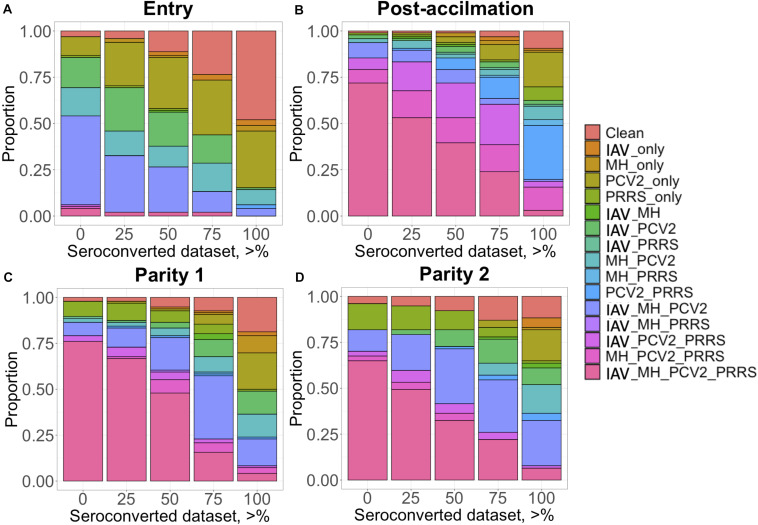
Proportion (*y*-axis) of contemporary group (CG) considered seropositive when a minimum percentage of individuals within this CG is positive (*x*-axis) for each time point: **(A)** Entry; **(B)** post-acclimation; **(C)** at Parity 1, and **(D)** at Parity 2. The colors represent the status of the individuals: clean, when free of any infectious diseases; IAV, influenza A virus; MH, *Mycoplasma hyopneumoniae;* PCV2, porcine circovirus type 2; and PRRS, Porcine Reproductive and Respiratory Syndrome; and all possible combinations of these infectious diseases.

The proportion of positive animals in each dataset is shown in Table 2. For IAV, MH, and PRRSV, most (i.e., >50%) of the CG had at least one seropositive animal after Post-acclimation and during P1 and P2, while for PCV2, most of the CG were seropositive at entry. Animals came from a high-biosecurity level (multiplier herd) and entered commercial farms where they were mixed with other pigs on the farm and had contact with diverse pathogens. Therefore, the lower proportion of seropositive animals at entry was expected. However, while for PRRSV the higher proportion of CG with at least one seropositive animal occurred at Post-acclimation, for IAV, MH, and PCV2, the proportion of CG with at least one seropositive animal was higher at P1 and P2. For APP, the proportion of CG with at least one seropositive animal was low at all time-points (0 to 21% of CG). There is little information on the actual prevalence of APP infection in the literature; however, a prevalence of 11% for pigs showing pleuritis in Canada has been reported (Amory et al., 2007), which may reflect the low incidence of this pathogen across these farms. There was also evidence of co-exposure during different time-points of the study (Figure 1). At entry, co-exposure (natural infection or vaccination) with IAV, MH, and PCV2 was the most common (47.9%). After the acclimation period, PRRSV became more prevalent in the co-exposure and 71.8% of CG were seropositive for IAV, MH, PCV2, and PRRSV. This co-exposure persisted to P1 (76.0%) and P2 (64.9%). If we consider only the CG with all the animals seroconverted (i.e., 100% SCD), PCV2 only or co-exposure with PCV2 and PRRSV were the most common (≥16.8%) at all time-points.

### Genetic Parameters

Heritability estimates (h^2^) for each trait for each dataset are presented in Table 3. For IAV, MH, and PCV2, h^2^ estimates for these traits were low to moderate, ranging from <0.01 ± 0.05 (PCV2 at P1) to 0.76 ± 0.07 (PCV2 at entry, 100% SCD). In general, h^2^ estimates numerically increased for datasets with a higher proportion of seropositive animals. This trend was more evident at entry. In contrast, APP serotypes had overall greater h^2^ estimates; APP10 showed the highest average h^2^ estimate (∼0.25), peaking at P2 (h^2^ = 0.38 ± 0.08), while APP2 had overall the lowest estimate (∼0.06). Among all traits analyzed, APP_mean_ had the highest average h^2^ estimate, ranging from 0.29 ± 0.06 at P1 to 0.55 ± 0.07 at P2. For overall Ab response (MEAN), h^2^ estimates ranged from low (0.08 ± 0.05; P2) to moderate (0.39 ± 0.5; post-acclimation). Overall, results indicate that selection for Ab response to some of these infectious pathogens is possible, depending on the time of collection.

**TABLE 3:** Heritability estimates^1^ of antibody response to common infectious pathogens^2^ in pigs by time-point.

**Traits^3^**	**%^4^**	**Entry**	**Post-accl^5^**	**Parity 1**	**Parity 2**
IAV	0	0.07 (0.04)	0.05 (0.04)	0.02 (0.04)	0.15 (0.07)
	25	0.11 (0.06)	0.08 (0.05)	< 0.01(0.04)	0.14 (0.07)
	50	0.18 (0.07)	0.10 (0.05)	0.05 (0.05)	0.14 (0.07)
	75	0.46 (0.10)	0.09 (0.05)	0.07 (0.05)	0.01 (0.05)
MH	0	0.19 (0.05)	0.15 (0.04)	0.17 (0.05)	0.10 (0.06)
	25	0.27 (0.08)	0.18 (0.05)	0.19 (0.05)	0.12 (0.07)
	50	0.27 (0.09)	0.19 (0.06)	0.21 (0.06)	0.11 (0.07)
	75	0.31 (0.11)	0.20 (0.07)	0.19 (0.06)	0.13 (0.07)
	100	0.31 (0.17)	0.21 (0.10)	0.12 (0.10)	0.14 (0.11)
PCV2	0	0.10 (0.04)	0.13 (0.05)	< 0.01(0.04)	0.02 (0.03)
	25	0.10 (0.04)	0.13 (0.05)	< 0.01(0.04)	0.02 (0.03)
	50	0.11 (0.05)	0.13 (0.05)	< 0.01(0.04)	0.02 (0.03)
	75	0.15 (0.06)	0.13 (0.05)	< 0.01(0.04)	0.02 (0.03)
	100	0.76 (0.07)	0.02 (0.04)	< 0.01(0.05)	0.02 (0.04)
APP1	0	0.29 (0.05)	0.16 (0.04)	0.14 (0.05)	0.30 (0.07)
APP2	0	0.13 (0.04)	0.11 (0.04)	< 0.01(0.04)	0.01 (0.05)
APP3	0	0.25 (0.04)	0.27 (0.05)	0.10 (0.04)	0.14 (0.06)
APP5	0	0.22 (0.04)	0.30 (0.05)	0.03 (0.04)	0.10 (0.06)
APP7	0	0.10 (0.04)	0.40 (0.05)	< 0.01(0.01)	0.25 (0.07)
APP10	0	0.21 (0.04)	0.24 (0.05)	0.17 (0.05)	0.38 (0.08)
APP12	0	0.22 (0.04)	0.27 (0.05)	0.22 (0.05)	0.19 (0.07)
APP13	0	0.25 (0.04)	0.24 (0.05)	0.15 (0.05)	0.31 (0.07)
APP_mean_	0	0.37 (0.05)	0.38 (0.05)	0.29 (0.06)	0.55 (0.07)
MEAN	0	0.32 (0.05)	0.39 (0.05)	0.14 (0.05)	0.08 (0.05)

*^1^Standard error (SE) within parenthesis. ^2^Antibody response to Porcine Reproductive and Respiratory Syndrome is not being showed as it has been previously published by Serão et al. (2016). ^3^Traits: IAV, antibody response to influenza A virus; M, antibody response to *Mycoplasma hyopneumoniae*; PCV2, antibody response to porcine circovirus type 2; APP, antibody response to *Actinobacillus pleuropneumoniae* from different serotypes (represented by the different numbers; APP_*mean*_, mean of antibody response to all serotypes of APP; MEAN, mean of standardized antibody response to all infectious pathogens. ^4^Minimum percentage of seropositive animals within a contemporary group. ^5^Post-accl, post-acclimation.*

**FIGURE 2:**
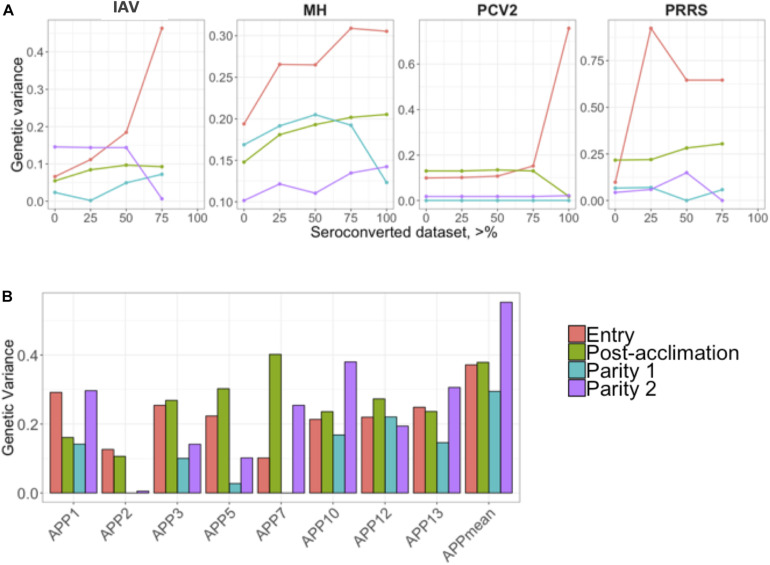
**(A)** Genetic variances for the seroconverted data for influenza A virus (IAV), *Mycoplasma hyopneumoniae* (MH), porcine circovirus type 2 (PCV2), and Porcine Reproductive and Respiratory Syndrome (PRRS); and **(B)** for *Actinobacillus pleuropneumoniae* (APP). The *y*-axis represents the genetic variances, and the *x*-axis represents the minimum % of positive animals within a contemporary group **(A)** or APP **(B)**. APP_mean_ represents the average of all serotypes of APP analyzed as the phenotype. The colors represent the different time-points of antibody response collection.

Estimates of additive genetic variance ($\sigma_{u}^{2}$) are presented in Figure 2. Similar to the h^2^ estimates for IAV, MH, and PCV2, estimates of $\sigma_{u}^{2}$ numerically increased as the proportion of positive animals increased in the dataset (Figure 2A). For APP, the estimate of $\sigma_{u}^{2}$ was numerically higher during P2. On average, APP2 had the lowest estimates of $\sigma_{u}^{2}$ and APP_mean_, the highest (Figure 2B).

**FIGURE 3:**
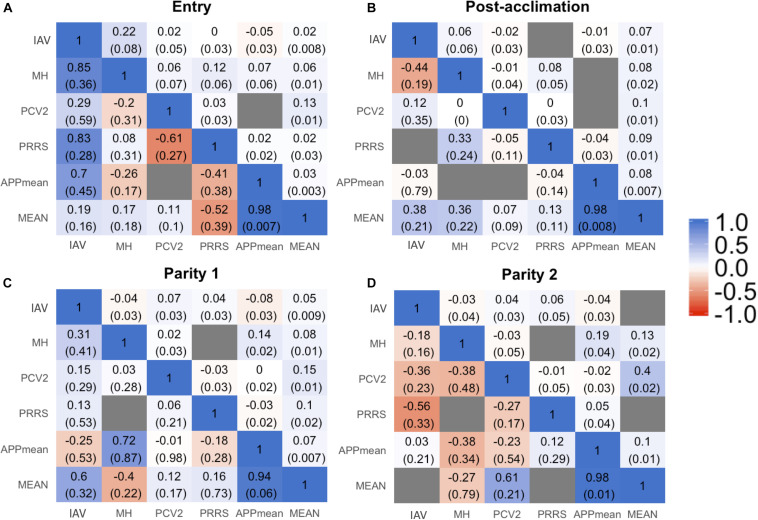
Genetic (lower triangular) and phenotypic (upper triangular) correlation between all the traits influenza A virus (IAV), porcine circovirus type 2 (PCV2), *Mycoplasma hyopneumoniae* (MH), porcine respiratory and reproductive syndrome (PRRS), average of all *Actinobacillus pleuropneumoniae* (APP_mean_), and overall mean of antibody response for all diseases (MEAN) at entry **(A)**, post-acclimation **(B),** Parity 1 **(C)**, and Parity 2 **(D)**. The seroconverted dataset with higher heritability within each time-point was used for these analyses. The blue color corresponds to positive correlation, the red color to negative correlation, and the gray color when the model did not converge.

Estimates of phenotypic and genetic correlations are shown in Figure 3. For all time-points, phenotypic correlations (Figure 3; upper diagonal) were generally low. The low phenotypic correlation associated with a low genetic correlation may indicate a low environmental correlation as well. Cases of low phenotypic correlation associated with a moderate to high genetic correlation may indicate a negative environmental correlation. Due to the low h^2^ of Ab response to the pathogens studied, we are reporting *r*_*G*_ estimates for the %SCD that had the highest h^2^ within each time-point and for APP_mean_. Estimates for each serotype of APP are available in Supplementary Figure 1. In summary, among APP, estimates of *r*_*G*_ were positive and moderate to high, ranging from 0.20 ± 0.19 to 0.99 ± 0.05. Between IAV and APP, estimates of *r*_*G*_ were negative at entry and post-acclimation. For the %SCD (Figure 3; lower diagonal), estimates of *r*_*G*_ of IAV with MH, PCV2, and PRRSV were consistently moderate to high and positive at all time-points, except for IAV and PCV2 at entry. Between IAV and APP_mean_, the estimate of *r*_*G*_ was moderate and negative at entry and post-acclimation but not at P2 (positive and low). Between PCV2 and PRRSV, the estimate of *r*_*G*_ was low to moderate and negative at all time-points. The estimate of *r*_*G*_ between APP_mean_ and PRRSV was positive at all time-points. Overall, *r*_*G*_estimates of APP_mean_ with all pathogens were consistent across time-points but among the other pathogens they were more variable, suggesting that genetic changes in one Ab trait may result in complex correlated responses to selection.

Estimates of *r*_*G*_ between time-points for a given pathogen are presented in Table 4. All estimates were positive and generally moderate to high for all traits, especially between consecutive time-points. APP1 showed the highest estimates of *r*_*G*_ between time points, ranging from 0.71 ± 0.17 between entry and P1 to 0.99 ± 0.08 between P1 and P2. On average, APP_mean_ had the highest estimate of *r*_*G*_ (0.77) compared to IAV (0.60), MH (0.62), PCV2 (0.51), and MEAN (0.61). Overall, these results indicate that selection for increased Ab response at one time-point would increase Ab response at all time-points.

**TABLE 4:** Estimates^1^ of genetic correlations within antibody response to common infectious pathogens in pigs between time-points^2^.

**Traits^3^**	**Entry vs. Post-accl^2^**	**Entry vs. P1^2^**	**Entry vs. P2^2^**	**Post-accl vs. P1^2^**	**Post-accl vs. P2^2^**	**P1 vs. P2^2^**
IAV	0.75 (0.29)	0.94 (0.94)	<0.01 (0.44)	0.73 (0.36)	0.65 (0.45)	0.82 (0.43)
MH	0.68 (0.14)	0.48 (0.18)	0.19 (0.30)	0.55 (0.18)	0.63 (0.28)	1 (0.14)
PCV2	0.04 (0.28)	NC^4^	NC^4^	0.79 (0.49)	0.92 (0.5)	0.96 (1.91)
APP1	0.96 (0.07)	0.71 (0.17)	0.82 (0.13)	0.91 (0.17)	0.99 (0.12)	0.99 (0.08)
APP2	0.90 (0.22)	NC^4^	NC^4^	NC^4^	NC^4^	NC^4^
APP3	0.95 (0.06)	0.55 (0.20)	0.54 (0.19)	0.61 (0.19)	0.64 (0.16)	0.85 (0.25)
APP5	0.74 (0.08)	0.86 (0.20)	0.87 (0.20)	0.8 (0.22)	0.93 (0.15)	0.69 (0.50)
APP7	0.97 (0.08)	0.67 (0.39)	0.62 (0.26)	0.95 (0.31)	0.33 (0.16)	0.84 (0.34)
APP10	1.00 (0.07)	0.53 (0.16)	0.64 (0.14)	0.63 (0.14)	0.85 (0.11)	0.92 (0.11)
APP12	0.97 (0.05)	0.66 (0.17)	0.08 (0.23)	0.44 (0.16)	0.29 (0.2)	0.81 (0.17)
APP13	0.96 (0.07)	0.16 (0.18)	0.59 (0.14)	0.46 (0.17)	0.81 (0.12)	0.95 (0.12)
APP_mean_	0.99 (0.04)	0.57 (0.11)	0.64 (0.09)	0.70 (0.10)	0.77 (0.07)	0.97 (0.05)
MEAN	0.91 (0.05)	0.73 (0.15)	0.52 (0.23)	0.77 (0.13)	0.32 (0.22)	0.41 (0.30)

*^1^Standard error (SE) within parenthesis. ^2^Post-accl, post-acclimation; P1, Parity 1; P2, Parity 2. ^3^Traits: IAV, antibody response to influenza A virus; M, antibody response to *Mycoplasma hyopneumoniae*; PCV2, antibody response to porcine circovirus type 2; APP, antibody response to *Actinobacillus pleuropneumoniae* from different serotypes (represented by the different numbers; APP_*mean*_, mean of antibody response to all serotypes of APP; MEAN, mean of standardized antibody response to all infectious pathogens. ^4^NC, model did not converge.*

### Genome-Wide Association Studies (GWAS)

Genomic regions that explained at least 1% of TGVM and that had a PPI > 0.7 are presented in Table 5. Within each analysis window, we selected the SNP that explained most of the TGVM and fitted the SNP individually in a total of 1-Mb window to estimate the genetic variance explained by that specific SNP (Supplementary Table 1). Several QTL were identified for APP serotypes, with most of them at entry and post-acclimation. Many of the identified regions described below included several candidate genes. For APP3 at entry, we identified 4 QTL on *Sus scrofa* chromosomes (SSC) 8, 9, 12, and 14 (2 Mb). The same QTL on SSC 14 was identified at post-acclimation. For APP5 at entry, 6 QTL on SSC 1, 4, 6, 9, and 13 and for at post-acclimation 5 QTL were located on SSC 2, 14, 6, and 8. For APP7 at post-acclimation, 2 QTL were identified on SSC 6 and 14. For APP 10 at entry, 1 QTL was identified on SSC 16. For APP13 at entry, there were 2 QTL on SSC 1 and 9; at post-acclimation, 2 QTL on SSC 14 and 16; and at P2, 2 QTL on SSC 6 and 7. For APP_mean_, there was 1 QTL on SSC 6 at entry; 3 QTL on SSC 7, 11, and 19 at post-acclimation; and 2 QTL at P2 on SSC 6 and 12. No QTL was identified for the other traits. The region on SSC14 (2 Mb) was associated with four different serotypes at entry and post-acclimation, suggesting that this is a key pleiotropic region associated with Ab response to APP.

**TABLE 5:** Percentage of total genetic variance explained for by markers (% TGVM) within a 1-Mb window for Ab response traits with significant QTLs using a threshold of 1% TGVM and posterior probability of inclusion (PPI) of 0.70.

**Traits^1^**	**Time-point**	**SSC^2^**	**Position (Mb)**	**Number of SNPs**	**% TGVM**	**PPI**
APP3	Entry	8	32	19	2.5	0.86
		14	2	12	1.9	0.88
		9	121	14	1.9	0.82
		12	47	11	1.2	0.72
APP3	Post-acclimation	14	2	12	3.9	0.96
APP5	Entry	9	6	37	1.7	0.93
		13	30	13	1.6	0.74
		1	58	24	1.5	0.72
		6	97	26	1.5	0.82
		4	63	21	1.3	0.75
		1	108	14	1.3	0.70
APP5	Post-acclimation	2	129	21	2.0	0.84
		14	2	12	1.9	0.86
		6	137	15	1.5	0.82
		8	125	15	1.4	0.86
		8	11	26	1.4	0.72
APP7	Post-acclimation	6	157	8	8.8	0.94
		14	2	12	4.3	0.95
APP10	Entry	16	68	23	3.1	0.77
APP13	Entry	1	58	24	5.7	0.89
		9	121	14	3.5	0.92
APP13	Post-acclimation	14	2	12	2.0	0.74
		16	73	22	1.7	0.70
APP13	Parity 2	6	81	23	1.8	0.74
		7	92	26	1.6	0.77
APP_mean_	Entry	6	79	20	3.5	0.84
APP_mean_	Post-acclimation	X	113	12	6.2	0.99
		11	61	20	4.6	0.95
		7	74	16	3.0	0.72
APP_mean_	Parity 2	6	93	13	2.7	0.80
		12	2	20	2.2	0.77

*^1^Traits, antibody response to APP, *Actinobacillus pleuropneumoniae* from different serotypes (represented by the different numbers); APP_*mean*_, mean of antibody response to all serotypes of APP. ^2^SSC, *Sus scrofa* chromosome.*

### Genomic Prediction Accuracies

**FIGURE 4:**
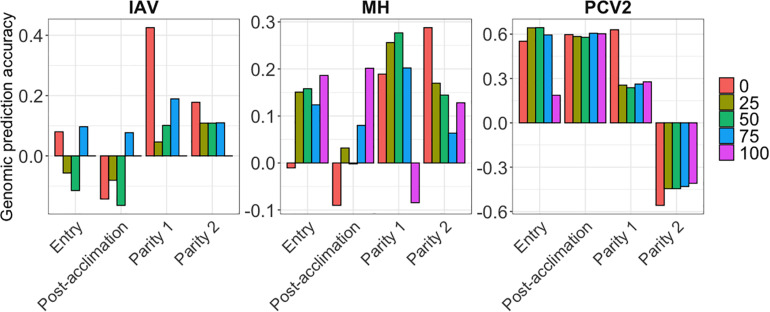
Genomic prediction accuracies (*y*-axis) for the seroconverted data for influenza A virus (IAV), *Mycoplasma hyopneumoniae* (MH), and porcine circovirus type 2 (PCV2) using BayesB. The *x*-axis represents the different time-points of data collection. The colors correspond to the minimum % of seropositive animals within a contemporary group.

Genomic prediction results are presented in Figure 4 for MH, IAV, and PCV2, using BayesB. AGP for IAV were low at all time points and all %SCD, except for 0% SCD at P1, ranging from −0.16 (post-acclimation) to 0.42 (P1). For MH, AGP were also low, ranging from −0.09 (post-acclimation) to 0.28 (P2). In contrast, PCV2 had the highest AGP among all pathogens at entry and post-acclimation, reaching 0.60 and 0.64, respectively. For PCV2 at P2, AGP were very low and negative, ranging from −0.55 (0% SCD) to −0.40 (100% SCD). Among methods evaluated, BayesB and BayesC had slightly higher accuracy than BayesC0. All results are compiled in Supplementary Table 2.

**FIGURE 5:**
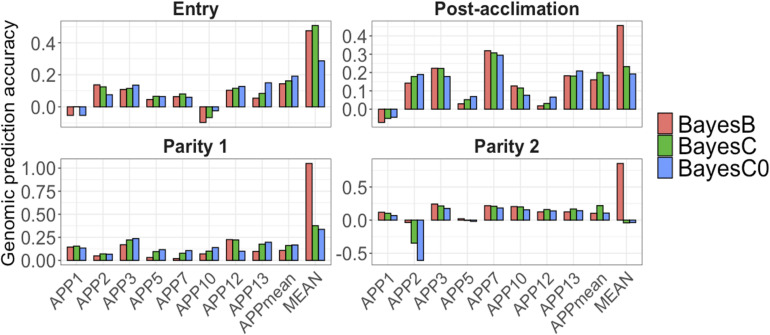
Genomic prediction accuracies (*y*-axis) for *Actinobacillus pleuropneumoniae* (APP1, 2, 3, 5, 7, 10, 12, and 13), average of all serotypes of APP (APP_mean_), and average of antibody responses for all diseases (MEAN). The *x*-axis represents the different time-points of data collection. The colors correspond to the Bayesian method used. Note the different scales of *y*-axis for each time-point to enhance visualization.

For all APP, AGP were low at all time-points (Figure 5). At each point, average AGP for APP using BayesB were 0.04 at entry, and 0.10 at post-acclimation, P1, and P2. Among the APP, APP7 showed the highest AGP (up to 0.31 at post-acclimation) and APP10, the lowest (up to −0.09 at entry). For APP_mean_, AGP ranged from 0.10 (P1 and P2) to 0.16 (post-acclimation). When analyzing all serology traits together, the AGP for MEAN were moderate to high (Figure 5), ranging from 0.45 (post-acclimation) to 1.05 (P1). Overall, these results indicate that genomic prediction for Ab response is possible, but results vary among traits and time-points.

## Discussion

In this study, we performed genetic and genomic analyses of Ab response to common infectious pathogens in pigs (IAV, MH, PCV2, and APP) that, along with PRRSV, have been identified as the main agents causing porcine respiratory disease complex, which causes considerable economic losses in the swine industry (Thacker et al., 2001). Few studies are available in the literature regarding the genetic basis of Ab response to these pathogens, especially for IAV and APP. Genetic parameters, GWAS, and genomic prediction accuracies for PRRSV using the same population from this study have been previously reported (Serão et al., 2016). Therefore, in this study, we focused on the relationship between Ab response to PRRSV with Ab response to all other pathogens. It is important to notice that none of the animals in this experiment were artificially inoculated with any of these pathogens. Also, different types of vaccination were used in some of the farms included in this study. However, limited information was available for these, including confirmation on whether these protocols were used for the animals in this study. However, using modified live vaccines is expected to generate similar humoral immune responses to wild-type infection [example for PRRSV (Montaner-Tarbes et al., 2019)]. For the %SCD, the increase in the proportion of seroconverted animals was confounded with a decrease in sample size, and the latter has been previously shown to result in decreased AGP for Ab response to PRRSV in this population (Serão et al., 2016). Moreover, the exact day of blood sample collection for Ab response measurement is uncertain but was confounded with CG. Therefore, the effect of CG should adjust for this effect in this dataset. Other factors, such as diet, management, season, and others, were also confounded in the study. However, differences in diets are not expected to affect Ab response to pathogens and most likely would not affect the conclusions (Pujols et al., 2016; Schweer et al., 2018; Colpoys et al., 2020). Nonetheless, these potential effects were captured by including the fixed effect of CG in the model. Despite these limitations of this study, this work provides genomic analyses, including estimates of h^2^ and *r*_*G*_, and identified regions with the potential to be used for genomic selection for an improved immune response to pathogens in commercial gilts and sows. With the increased pressure by society for animal welfare, the industry has been motivated to investigate resilience traits. Antibody response to specific diseases could reflect the overall immune status of the individual, and although not all pathogens stimulate the similar humoral immune response, it is an important trait to be investigated. Our hypothesis is that selection for an improved Ab response to pathogens is followed by selection for better immune defense of the organism when the animal is facing diseases and, consequently, lesser disturbance of the performance in healthy challenging environments (i.e., more resilience).

Summarizing, although there were some limitations on the study, such as the lack of confirmation of whether animals were vaccinated and/or naturally infected, the existence of several confounding factors (e.g., diet, management, and others), and the lack of information on how long after the exposure the Ab was collected, the advantages prevailed over the disadvantages. The applicability of these results in commercial settings, the possibility of using crossbred performance for selection, identifying novel traits for selection of resilience in pigs, and the use of relevant pathogens common in the swine production are valuable to the pig industry.

### Genetic Parameters

To the best of our knowledge, this is the first study reporting genetic parameters for Ab response to MH, PCV2, and APP in gilts and sows. Estimates of h^2^ were low to moderate for all Ab responses analyzed. Estimates of h^2^ for Ab response to IAV were, in general, low, except at entry (0.46), when 75% of the animals within a CG were positive. Previously, an estimate of h^2^ for Ab response to IAV of 0.37 has been reported in F1 (Landrace × Large White) piglets (∼76 days old) after vaccination (two doses) to IAV (Zanella et al., 2015). It is important to note, however, that our data were collected across multiple CGs. Thus, multiple confounding effects could explain the lower estimates in our study, such as the fact that not all animals in our study were positive to IAV. Our data is further complicated by the uncertain exposure of the animals, i.e., whether they were naturally infected or vaccinated, the number of vaccination doses received, and the age of the animals when Ab response was measured.

Among all individual pathogens evaluated in our study, MH, in general, presented the highest h^2^ of Ab response. Okamura et al. (2012) reported an h^2^ estimate of 0.23 for lesion score of mycoplasma pneumonia measured in slaughtered pigs that were experimentally inoculated with MH. In their study, 59% of the animals were considered positive based on lung lesions (Okamura et al., 2012). Their results are similar to ours, where we obtained an average estimate of h^2^ of 0.20 for the 50% seroconverted dataset. In another study, also analyzing the score of mycoplasma pneumonia of swine based on lung lesions in slaughtered pigs after vaccination at 6 and 8 weeks of age, the estimate of h^2^ was 0.09 (Sato et al., 2016). These two results are not directly comparable with ours since the animals were experimentally infected and the phenotype analyzed was not the same. However, these are, to the best of our knowledge, the only reports on the genetic basis of MH in pigs available in the literature.

For PCV2, h^2^ estimates were low at all time-points, except for CG for which all animals were seropositive at entry. Although not analyzing the same trait, Dunkelberger et al. (2017) reported an h^2^ estimate of 0.09 for viral load of PCV2 after co-natural infection and vaccination to PRRSV, where 100% was experimentally infected with both pathogens. On the other hand, Walker et al. (2018) reported a high h^2^ of 0.64 for PCV2 viral load, with a major QTL located on the MHC class II region. In addition, Bates et al. (2009) reported an estimate of 0.16 for clinical score for PCV2. Although we obtained a very high h^2^ estimate for PCV2 Ab response at entry including CG where all animals had seroconverted for PCV2, these results suggested that response to PCV2 is highly influenced by the environment and less determined by host genetics. Thus, in order to use PCV2 Ab response for genetic selection, all animals must be seroconverted when Ab response data is collected.

For APP, estimates of h^2^ were low to moderate, ranging from <0.01 (APP2 and APP7 at P2) to 0.40 (APP7 at post-acclimation). In general, APP13 showed higher h^2^ estimates (average of 0.25). Although all APP serotypes can cause the same disease, some serotypes may be more virulent than others (Bossé et al., 2002) and cross-protection between serotypes is limited (Haesebrouck et al., 1997). The incidence of APP seroconversion was very low for all APP serotypes, which may explain the low h^2^ of APP Ab response. Averaging the Ab response, overall APP serotypes resulted in a substantial increase in estimates of $\sigma_{u}^{2}$ and h^2^, which may occur because of the variation in Ab response from each individual to each serotype. Similar to APP_mean_, the h^2^ estimate for overall MEAN was also higher than estimates of h^2^ for Ab response to individual pathogens, especially at entry and post-acclimation. The higher h^2^ for the overall Ab response across pathogens (MEAN) indicates that selection for this trait in sows under healthy challenge may be more successful than selection for Ab response for specific pathogens.

In general, an increase in the proportion of positive individuals in the dataset for IAV, MH, and PCV2 increased the estimate of $\sigma_{u}^{2}$ for Ab response, as expected (Bishop and Woolliams, 2010), and in an increase in the estimate of h^2^. Similarly, the estimate of $\sigma_{u}^{2}$ of Ab response to APP was higher at P1 and P2 (except for APP2 and APP5), which were also the time-points with higher proportions of positive animals. Similar results were previously reported for PRRSV using samples from this same study (Serão et al., 2016). The low to high h^2^ estimates indicate a great variation in the practicability of the use of Ab response traits in commercial swine populations for genetic selection purposes. Altogether, these results indicate that, in order to obtain high genetic variation for Ab response to common infectious pathogens in commercial sows, exposure to these pathogens must happen, via either vaccination and/or natural infection.

It is well known that the infection of an individual by immunosuppressive pathogens, such as PPRSV, weakens its immune system, favoring the entry or multiplication of a second pathogen. For instance, studies have shown that co-infection between some of these agents frequently intensifies the clinical signs of the diseases (Thacker et al., 2001; Dunkelberger et al., 2017). In this study, Ab response to IAV had positive moderate to high estimates of *r*_*G*_ with Ab response to MH and PRRSV at entry, when the proportion of positives for IAV, PCV2, and MH was higher. At post-acclimation, this relationship became negative, coinciding with the increase in the number of positives for PRRSV. A previous study has shown low interactions between MH and IAV (Thacker et al., 2001) such that co-infection with these two pathogens did not intensify the clinical signs from the other. IAV seems to be easily eliminated from the organism by neutralizing antibodies, and there is little or no interference with the activation of the immune system to fight against other pathogens (Holzer et al., 2019). However, the introduction of PRRSV caused a change in the direction of the *r*_*G*_ between Ab response to MH and PRRSV. Conversely, the estimate of the *r*_*G*_ between PRRSV and PCV2 was consistently negative. In commercial settings, co-infection with these two pathogens is common (Engle et al., 2014). Dunkelberger et al. (2017) reported a *r*_*G*_of 0.27 (0.08) between PRRSV and PCV2 viral load in pigs that were PRRSV-vaccinated and co-infected with both pathogens, but a near-zero *r*_*G*_ in non-PRRSV vaccinated pigs [*r*_*G*_ = 0.04 (0.09)]. However, our estimates for *r*_*G*_ for Ab response to PCV2 and PRRSV were negative, suggesting that the immune response to one pathogen is compromised by co-infection with the other pathogen. PCV2 natural infection tends to inhibit innate immune response, which is the initial response to fight against PRRSV infection (Montaner-Tarbes et al., 2019). If infection by one pathogen weakens the immune response to another pathogen, this may cause a negative *r*_*G*_ of Ab responses to both pathogens. These results indicate that selecting for increased Ab response to PCV2 could result in a small reduction in Ab response to PRRSV.

At entry, the estimate of *r*_*G*_ of APP_mean_ was moderate to high positive with IAV and moderate negative with MH. To the best of our knowledge, no reports have shown an association between APP infection and predisposition of viral or bacterial infections, although an increase in the incidence of pleuropneumonia has been associated with increased environmental stress (Bossé et al., 2002). APP is rapidly eliminated by the innate immune response with little interference in the response to other pathogens (Sato et al., 2016), which may explain the positive *r*_*G*_ between APP_mean_ and MH found in our study. The *r*_*G*_ estimate of MEAN with APP_mean_ was positive and high at all time points, which may be because the serotypes of APP composed most of the Ab responses used to calculate MEAN, in addition to Ab responses to different APP serotypes showing high positive *r*_*G*_ with each other. This *r*_*G*_ was also positive (although sometimes low) with IAV and PCV2. The *r*_*G*_ of MEAN with PRRSV at entry, and with MH at P1 and P2 were negative, indicating that selection for MEAN is possible but should be done with care. Genetic selection over total Ab response is expected to have a correlated response with antibody response to individual pathogens. Thus, it can affect the genetic capacity of the organism to deal with these pathogens, which should be taken in consideration when selecting for immune response-related traits.

The results discussed above were obtained using the 100% SCD for MH, IAV, and PCV2, which had the highest h^2^ estimates for Ab response to each pathogen within a time-point. We also evaluated the *r*_*G*_ and *r*_*P*_ for the 0% SCD, which had low h^2^ and *r*_*G*_ and obtained an overall similar direction, but lower estimates and greater SE (Supplementary Figure 1).

Altogether, these results suggest that genetic progress for direct selection on Ab response traits depends on several factors, such as timing, level of co-exposure, and the number of seroconverted animals. The different extent in innate vs. humoral immune response may also have an effect on the genetic parameters of this traits (Flori et al., 2011; Mangino et al., 2017) as they are related to each other, and one can limit or stimulate the action of the other. Nonetheless, we observed substantial genetic variation for Ab response in this dataset, indicating that the use of specific time-points with a high proportion of seroconverted animals could be an efficient strategy to improve Ab response in commercial sows.

### Genome-Wide Association Studies (GWAS)

Several QTL were identified for Ab response to APP at different time-points but not for other infectious pathogens in pigs. APP is highly contagious and can cause pleuropneumonia in pigs. The existence of many APP serotypes can limit its prevention and effective cure (Liu et al., 2017). The difference in virulence of different serotypes is caused mainly by the presence of different toxins and amounts of lipopolysaccharides (LPS) on the surface of the microorganism (Bossé et al., 2002). Therefore, identifying genomic regions associated with Ab response to serotypes of APP could help the use of this trait in selection purposes and a better understanding of the genetic component. Although it has been reported that cross-protection between the APP is limited, the QTL on SSC 14 for 4 of the serotypes suggests the presence of a pleotropic gene in this region. This QTL on SSC 14 at 2 Mb was found to be associated with Ab response to serotypes 3, 5, 7, and 13, especially at post-acclimation, mainly by two SNP, ALGA0074334 and H3GA0038333. This region contains the spleen associated tyrosine kinase (*SYK*) gene which has been associated with surface immunoglobulin (Ig)M complexes and appears to stimulate the signaling cascade in B lymphocytes via an antigen receptor (Müller et al., 1994; Seow et al., 2002). APP antigen stimulates the Ab-mediated immune response, which is produced by B lymphocytes (Appleyard et al., 2002). Interestingly, this same region has also been associated with total number of piglets born in Yorkshire (Do et al., 2018), indicating that selection for Ab response to APP may be associated with indirect selection for resilience in sows, measured as the capacity of maintaining reproductive performance in a disease-challenge environment.

For APP3 at entry, another 3 QTL were identified on SSC 8 (32 Mb), 9 (121 Mb), and 12 (47 Mb). On SSC 8, the ubiquitin C-terminal hydrolase L1 (*UCHL1*) gene has been reported to affect the ovulation rate in the pig (He et al., 2017). A potential candidate gene in the region on SSC 9 (121 Mb) is sterol O-acyltransferase 1 (*SOAT1*), which has been shown to be upregulated in pigs infected with APP7 in comparison to healthy animals (Reiner et al., 2014a). The QTL identified for APP3 at entry in the region on SSC 12 (47 Mb) has previously been associated with survival and clinical signs after challenge with APP7 in an F2 swine population (Reiner et al., 2014b). This region harbors the vitronectin (*VTN*) and fucosyltransferase 2 (*FUT2*) genes, which were less expressed in the liver of healthy animals compared to pigs infected with APP (Skovgaard et al., 2010).

For APP5 at entry, the regions on SSC 9 (6 Mb), 13 (30 Mb), and 4 (63 Mb) include genes that were previously found to be down- (tripartite motif-containing 55, *TRIM55*) and upregulated (diacylglycerol O-acyltransferase 2, *DGAT2*; and uroplakin 1B, *UPK1B*) in pigs infected with APP7 (Reiner et al., 2014a). In addition, *UPK1B* is part of the innate immune system and has been associated with urinary tract infection by gram-negative bacteria in humans (Ertan et al., 2010). Furthermore, the region on SSC 13 (30 Mb) includes several immune genes associated with chemokines, such as the c–c motif chemokine receptor 9 (*CCR9*), c–x–c motif chemokine receptor 6 (*CXCR6*), c–c motif chemokine receptor 2 (*CCR2*) and 5 (*CCR5*), and c–c motif chemokine receptor-like 2 (*CCRL2*). These genes are most related to cytokine–cytokine receptor interactions. Of those, *CCRL2* has recently been implicated in the regulation of reproductive functions in pigs (Gudelska et al., 2020). The region on SSC 4 (63 Mb) has also previously been associated with number of piglets mummified in a large White population (Wu et al., 2019). The region on SSC 8 (125 Mb) contains the secreted phosphoprotein 1 (*SPP1*) gene, which was less expressed in the liver of healthy animals compared to pigs infected with APP (Skovgaard et al., 2010).

For APP5 at post-acclimation, besides the region on SSC 14 (2 Mb), the region on SSC 2 (129 Mb) has been previously associated with APP natural infection in swine (Tsai et al., 2011). This region includes the CD molecule (*CD14*) gene, which along with lymphocyte antigen 96 (*MD2*) and toll-like receptor 4 (*TLR4*), mediates the innate immune response to bacterial LPS, leading to NF-κB activation, cytokine secretion, and the inflammatory response (Tsai et al., 2011). LPS is one of the main virulence factors of APP, making *CD14* a potential candidate gene (Reiner et al., 2014b). Besides the QTL for APP7 at post-acclimation on SSC 14 (2 Mb), another QTL on SSC 6 (157 Mb) was identified, where the transmembrane protein 59 (*TMEM59*) is located. This gene encodes for a protein that has been shown to regulate autophagy in response to *Staphylococcus aureus* infection.

For APP13 at P2, the region on SSC 6 (81 Mb) contains several complement genes, such as the complement C1q (*C1Q*) A chain (*C1QA*), C1Q B chain (*C1QB*), and C1Q C chain (*C1QC*). Complement activation is one of the mechanisms of defense stimulated by APP (Bossé et al., 2002), making these genes potential candidates associated with Ab response to this pathogen. The region on SSC 7 (92 Mb) has also been associated with teat number in swine, an important reproductive trait in pigs (Ding et al., 2009). For APP_mean_, 2 of the QTL identified at post-acclimation, on SSC X (113 Mb) and 11 (61 Mb), have previously been associated with IgG2 and eosinophil counts, respectively, in Meishan vs. Pietrain pigs infected with *Sarcocystis* sp. (Reiner et al., 2007). The region on SSC 12 (2 Mb) has previously been associated with the sonographic score (based on reflections of high-frequency sound waves) of APP in Hampshire vs. Landrace pigs after challenge with APP7 (Reiner et al., 2014b). The region on SSC 7 (74 Mb) harbors the T-cell receptor alpha locus (*TCRA*), interferon-stimulated transcription factor 3 gamma (*IRF9*), and ribonuclease RNase A family 4 (*ANG*) genes, which have previously been associated with APP natural infection (Skovgaard et al., 2010). Summarizing, most of the candidate genes associated with APP seems to be associated with NF-Kb activation and the complement system. Interestingly, NF-Kb is increased during PRRSV infection (Guo et al., 2017), which can be an important factor during co-infection with these two pathogens. Furthermore, the complement system is part of the innate immune response that influences an acquired immune response (Dempsey et al., 1996), and thus, genes regulating this system may be involved in the genetic control of the antibody response to APP.

Interestingly, several of the regions identified for APP are associated with reproductive traits in pigs, such as number of pigs born, ovulation rate, and number of teats, indicating that the identified QTL for APP could be used for the improvement of resilience in commercial sows. The lack of QTL for the trait MEAN can be due to the dilution effect of some traits having major QTL and others not. Nonetheless, our results suggest that a larger part of the genetic variation for most infectious pathogens explained by several QTL with small effects.

### Genomic Prediction Accuracies

Several studies have exploited the use of immune-related traits, such as viral load, level of cytokines, and clinical signs to infectious diseases, for the selection of individuals with a better immune response (Wieland et al., 2004; Kaiser et al., 2005; Thompson-Crispi et al., 2014). However, few studies have focused on the acquired immune response. Serão et al. (2016), using part of the data used in the current study, suggested that Ab response to PRRSV after acclimation can be predicted across populations using SNP. They reported greater AGP when using SNP within the two major QTL for Ab response to PRRSV on SSC7 (30 and 130 Mb) compared to the rest of the genome. Sanglard et al. (2020) observed greater AGP for Ab response to PRRSV than in Serão et al. (2016) in PRRSV-vaccinated gilts from the same population. Ab response to Newcastle disease and avian influenza in chickens was studied by Liu et al. (2014), who reported moderate prediction accuracy for these traits. In our study, AGP ranged from very low to high, depending on the pathogen and time-point.

In general, BayesB is expected to have higher accuracy than BayesC0 in the presence major QTL since BayesB gives more emphasis to QTL with higher effect and shrinks the effect of the other SNP toward zero (Fernando and Garrick, 2013). This pattern was observed for some traits in our study, as we observed a higher accuracy with BayesB for APP10 at entry and APP3 at post-acclimation, both with identified major QTL. Nonetheless, not all traits followed this pattern. For example, we observed cases where no QTL was identified but still, BayesB performed better (such as for IAV, MH, PCV2, and MEAN) or a QTL was identified but BayesC0 performed better (such as APP_mean_). The performance of BayesC and BayesC0 relative to other methods depends on the actual distribution of the marker effects (Fernando and Garrick, 2013), which is unknown for the novel traits evaluated in this study.

For Ab response to IAV, the low AGP are in accordance with the fact that no QTL were identified for this trait across all time-points, and its low h^2^ estimates. For Ab response to MH at entry and post-acclimation, AGP were higher when using 100% SCD. This is in contrast to results by Serão et al. (2016) for Ab response to PRRSV, where lower AGP were observed with increasing %SCD. However, Serão et al. (2016) indicated that this may have been caused by a major reduction in the dataset analyzed with 100% SCD compared to 0% SCD (when AGP was the highest). In our study, however, the major reduction in the size of the dataset from 0% (2,355) to 100% SCD (564) did not seem to negatively impact the results. At P2, the AGP decreased as the %SCD increased (and the number of animals decreased), more similar to what was observed by Serão et al. (2016). For Ab response to PCV2, the AGP were moderate to high. This must have happened because of the very low h^2^ estimates for these traits since the division of the correlation between GEBV and adjusted phenotypes by the square root of the h^2^ is part of the calculation of AGP. In fact, the average correlations were quite low for PCV2, ranging from −0.04 (75% SCD) to −0.05 (0% SCD). Therefore, the high AGP found for PCV2 has little implication for selection purposes.

For all APP, although some QTL were identified for most serotypes, the AGP were low, including for APP_mean_, indicating a limitation for the use of this trait for selection purposes. Similar to h^2^, the low number of positive animals for APP may limit the genetic expression of these traits among the animals in this dataset; therefore, studies involving Ab response to vaccination or natural infection to APP should not be excluded from future works.

The AGP for MEAN were higher than for the other traits, especially at entry and post-acclimation. Although no major QTL was identified for these traits, the overall sum of small QTL effects captured by the markers suggests that genomic prediction can be used to identify animals with overall better acquired immune response to the pathogens included in this study. This may happen because the SNPs spread along the genome are capturing QTLs with small effects, resulting in overall greater accuracy, even in the absence of major QTLs. This corroborates that selection on the overall mean of Ab response to common pathogens may be more efficient than selection on Ab response to individual pathogens. These results suggest that the genomic predictive ability of most of these traits is limited, but some of them (i.e., MEAN) have the potential to be further explored.

## Conclusion

For the first time, the genetic basis of Ab response to a range of pathogens in pigs was explored in commercial sows. Differences in the Ab response exist for different pathogens; however, this trait may be still a proxy for resilience in commercial sows. Our results revealed that these traits have low intermediate heritabilities, with exception of APP_mean_ and MEAN. In addition, important genomic regions were identified for some APP serotypes. Most of the Ab response traits had low to moderate genomic predictive ability, especially when no QTL were identified. However, MEAN had moderate to high genomic prediction accuracies. These results suggest that genetic progress by selection on Ab response to these pathogens is possible but may be slow and that selection on the average Ab response to common pathogens in pigs may be an alternative strategy. The use of specific sample collection time-points can result in higher heritabilities, as well as datasets with a higher proportion of seroconverted animals, to increase the genetic variance. Some disadvantages such as the lack of confirmation of whether animals were vaccinated and/or infected with these pathogens, the existence of several confounding factors (e.g., diet, management, and others), and the lack of information on how long after the exposure the blood was collected and the course the pathogen could limit the interpretation of the results obtained. However, this variability is a strength for the application of these results in commercial settings, as the ability to test for infection with all of these pathogens may not be realistic. Other advantages of this study include the possibility of using crossbred performance for selection, the identification of novel traits for selection of resilience in pigs, the use of commercial populations reared in true commercial conditions, and the use of relevant pathogens that are easy to be measured. New studies including commercial performance, such as reproductive performance, are needed to better understand the relationship between Ab response to these pathogens and commercially important traits in the swine production.

## Members of the PigGen Canada Consortium

They participated in project and protocol development and implementation, coordinated the sources of sows and collection of associated data, and contributed to the project through regular discussions during execution of the gilt acclimation project: Mr. D. Vandenbroek and Mr. B. DeVries, Alliance Genetics Canada, St. Thomas, ON, Canada; Dr. N. Dion and Ms. S. Blanchette, AlphaGene, Saint-Hyacinthe, QC, Canada; Dr. T. Rathje, DNA Genetics, Columbus, NE, United States; Mr. M. Duggan, FastGenetics, Saskatoon, SK, Canada; Dr. R. Kemp, Genesus, London, ON, Canada; Dr. P. Charagu, Hypor, Regina, SK, Canada; and Dr. P. Mathur, Topigs Norsvin, Helvoirt, Netherlands.

## Data Availability Statement

The data that support the findings of this study are not publicly available. Data may be available from authors upon request and authorization from the company that generated the data.

## Ethics Statement

The animal study was reviewed and approved by the Canadian Council on Animal Care (2020).

## Author Contributions

LS performed the data analyses, interpreted the results, and drafted the manuscript. PC, JH, SB, GP, and JD developed the research project. BM coordinated the data collection. PW coordinated the database. LS, JD, and NS conceived the statistical analyses. LS and NS prepared the first draft of the manuscript. All authors contributed to the final manuscript, and read and approved the final manuscript.

## Conflict of Interest

The authors declare that the research was conducted in the absence of any commercial or financial relationships that could be construed as a potential conflict of interest.

## Footnotes

1 http://www.piggencanada.org/

## Supplementary Material

Supplementary Figure 1 Genetic (lower triangular) and phenotypic (upper triangular) correlation between all traits: influenza A virus (IAV), porcine circovirus type 2 (PCV2), *Mycoplasma hyopneumoniae* (MH), *Actinobacillus pleuropneumoniae* (APP), and porcine respiratory and reproductive syndrome (PRRS) at Entry (A), Post-acclimation (B), Parity 1 (C), and Parity 2 (D). The values between parenthesis correspond to the standard error of the correlation. The blue color corresponds to positive correlation, the red to negative correlation, and the gray indicate the lack of convergence of the model.

Supplementary Table 1 Percentage of total genetic variance explained for by markers (% TGVM) within a 1-Mb window for traits with significant QTLs using a threshold of 1% TGVM and posterior probability of inclusion (PPI) of 0.70 (before SNP selection). The SNP explaining most of the TGVM within a window was selected and the % TGVM explained by the SNP (% TGVM-SNP) was also estimated (after SNP selection).

Supplementary Table 2 Accuracies of genomic prediction.

## Abbreviations

Ab: antibody
AGP: accuracy of genomic prediction
APP: *Actinobacillus pleuropneumoniae*
CG: contemporary groups
GC: gene call
GRM: genomic relationship matrix
GWAS: genome-wide association studies
h^2^: heritability
IAV: influenza A virus of swine
MH: *Mycoplasma hyopneumoniae*
P1: parity 1
P2: parity 2
PCV2: porcine circovirus type 2
PRRS: porcine reproductive and respiratory syndrome
PRRSV: PRRS virus
QTL: quantitative trait loci
r_g_: genetic correlation
SCD: seroconverted datasets
SSC: chromosomes
TGVM: total genetic variance explained by the markers.
