# Supplementary figures and images for: Genomic Analysis of IgG Antibody Response to Common Pathogens in Commercial Sows in Health-Challenged Herds
Source: Front Genet. 2020 Oct 23;11:593804. doi: 10.3389/fgene.2020.593804 (PMC7646516; doi:10.3389/fgene.2020.593804)

Supplementary Figure 1


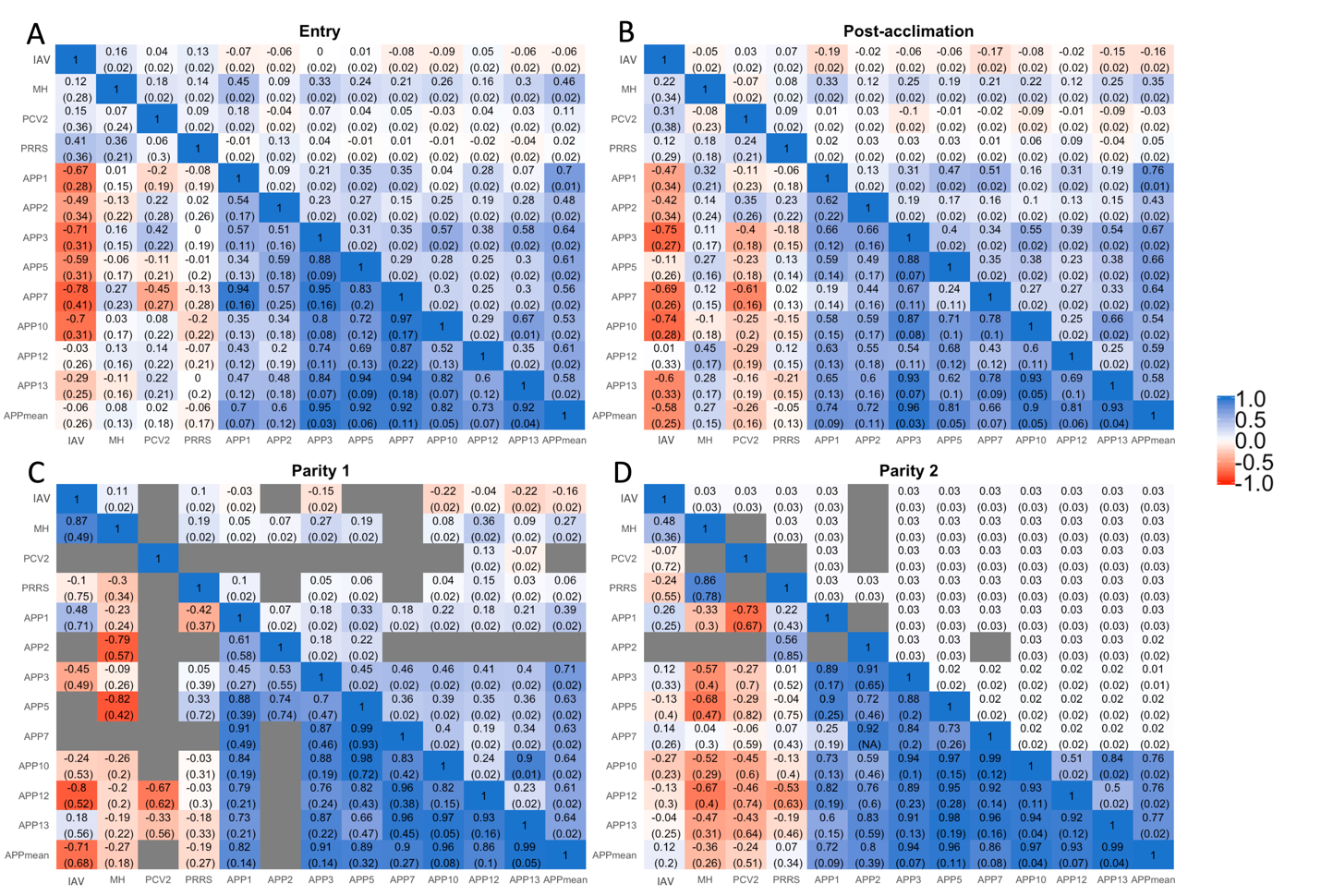

Supplement: Supplementary Figure 1 — Genetic (lower triangular) and phenotypic (upper triangular) correlation between all traits: influenza A virus (IAV), porcine circovirus type 2 (PCV2), Mycoplasma hyopneumoniae (MH), Actinobacillus pleuropneumoniae (APP), and porcine respiratory and reproductive syndrome (PRRS) at Entry (A), Post-acclimation (B), Parity 1 (C), and Parity 2 (D). The values between parenthesis correspond to the standard error of the correlation. The blue color corresponds to positive correlation, the red to negative correlation, and the gray indicate the lack of convergence of the model. [file Data_Sheet_1.docx]
